# Supplementary material for: Spatial Metrics of Tumour Vascular Organisation Predict Radiation Efficacy in a Computational Model
Source: PLoS Comput Biol. 2016 Jan 22;12(1):e1004712. doi: 10.1371/journal.pcbi.1004712 (PMC4723304; doi:10.1371/journal.pcbi.1004712)
Supplement: S1 Text — (PDF) [file pcbi.1004712.s002.pdf]

Supplemental Information for: Spatial metrics of tumour vascular organisation predict radiation efficacy in a computational model

Software S1

Please find attached a zipped folder containing java implementation of the mathematical model and MATLAB implementation of the analytic tools described in this study. A thoroughly documented example of how to run the code is provided in the file README.txt.

Text S1

In the following supplementary material, we: provide details of our numerical method of solution of the oxygen transport equation; check our parameter choices using dimensional analysis and some simple physical arguments; describe in detail our choice of oxygen update time step and stability requirements; perform a sensitivity analysis of the model parameters; provide details on the spatial statistic used in our study and its correlation to carrying capacity; and show the correlation and p-values for the plots in Fig 8.

Numerical solution. To solve equation (1) in the main text numerically, we discretise space and time by letting $t_k = k\Delta t$, where $k \in \mathbb{N}$, and $x_i = i\Delta x$ and $y_j = j\Delta x$ where $i, j \in \{1, \dots, N\}$ encode a square lattice of size $N \times N$, and Δt is the oxygen update time step. We approximate the oxygen concentration at time t_k and position x_i, y_j by $c_{i,j}^k \approx c(x_i, y_j, t_k)$. We use a central difference approximation for the Laplacian and thus approximate equation (1) in the main document by

$$\frac{c_{i,j}^{k+1} - c_{i,j}^k}{\Delta t} = \frac{D_C}{\Delta x^2}\left[c_{i+1,j}^k + c_{i-1,j}^k + c_{i,j+1}^k + c_{i,j-1}^k - 4c_{i,j}^k\right] - \left(f_c\right)_{i,j}^k, \tag{1}$$

where $\left(f_c\right)_{i,j}^k$ is the cell-specific oxygen consumption rate defined in equation (2) in the main text. Rearranging equation (1) to obtain a solution for $c_{i,j}^{k+1}$ gives

$$c_{i,j}^{k+1} = c_{i,j}^k\left(1 - 4\frac{D_C\Delta t}{\Delta x^2}\right) + \frac{D_C\Delta t}{\Delta x^2}\left(c_{i+1,j}^k + c_{i-1,j}^k + c_{i,j+1}^k + c_{i,j-1}^k\right) - \Delta t(f_c)_{i,j}^k. \tag{2}$$

At each oxygen time step, the oxygen concentration in a given lattice site is updated using equation (2). To impose the zero-flux boundary conditions we modify equation (2) in the cases where $i, j \in \{1, N\}$. For example, to calculate the oxygen concentration experienced by a cell at the left-hand boundary (i.e. $i = 1$) from outside the domain (i.e. $i = 0$) we discretise the no-flux boundary condition to obtain $c_{0,j}^k = c_{2,j}^k$, which we substitute into equation (2). Note that lattice sites occupied by vessels have fixed oxygen values (1 in the non-dimensional system), so we do not update them.

Parameter estimation. We assume that normal (brain) tissue has an approximate oxygen concentration of 35mmHg [1]. This value also agrees well with an estimate of background tissue oxygen concentration of $c_0 = 1.7 \times 10^{-8}$ mol cm^{-2} (4.25×10^{-13} mol cell^{-1}) taken from Anderson and colleagues [2] after using the ideal gas law, assuming body temperature of 310K, oxygen tension of 5300Pa [1] and cell volume of $125,000\mu$m^3.

As each of these parameters has been estimated from different sources, we will fine tune the basal oxygen consumption and physiologic vascular density for our specific case using a well-studied tumour spheroid example. We utilize the observation that the diffusion distance of oxygen to support cancer cells is approximately 10 cell diameters [3] and information from the literature concerning the ratio of cancer to normal oxygen consumption. To estimate the baseline oxygen consumption rate then, we begin with the value $r_c = 2.3 \times 10^{-16}$ mol cell^{-1} s^{-1} taken from an *in vitro* study of tumour spheroid growth [4] and then perform a virtual tumour spheroid assay (Fig. A) to fine tune the value for our model system.

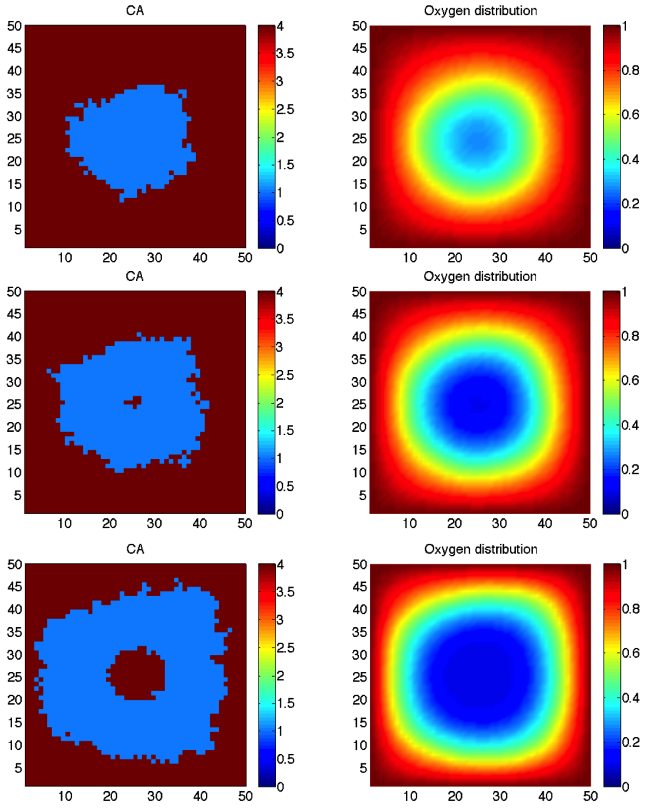

Figure A: Tumour growth in an avascular domain with oxygen diffusion from the outside displays characteristics of tumour 'spheroid' growth. Here, an otherwise empty domain is initiated with a single cancer cell. The oxygen at the edge of the domain is set to $c = 1$. Cells (left) and oxygen concentration (right) are plotted at three time points: before the onset of central necrosis (top), initiation of central necrosis (middle) and later in progression (bottom) when a nearly constant sized proliferative 'rim' is observed. From this calibration, we find that the maximal oxygen uptake rate most appropriate for our model is $r_c = 7.5 \times 10^{-4}$ s^{-1} which correlated with an approximate 10 cell diameter thickness.

Time scales and updates. The difference in time scales between the diffusion of oxygen and the proliferation of cells is taken into account by updating the continuous part of the model many times per cellular time step. This can become computationally expensive in this explicit scheme, and therefore, we seek to minimize this number. However, for stability, we require $\Delta t D_c/\Delta x^2 < 0.25$ [5]. We therefore choose $\Delta t D_c/\Delta^2 = 0.1$. In dimensional parameters, we then calculate

$$\Delta t = \frac{0.1(50 \times 10^{-4}\ \text{cm})^2}{1 \times 10^{-5}\ \text{cm}^2\ \text{s}^{-1}} = 0.25\ \text{s}, \tag{3}$$

which equates to approximately $230,400$ times per cell cycle based on the parameters chosen (see Table 1 in main text).While we assume the average cell cycle time to be $\tau = 16$ hours, it is well known that cells in tissues are not synchronized, and also that cell fate decisions such as apoptosis are made on shorter time scales. To model this heterogeneity in cell cycle time and to more accurately match the finer time scale associated with cell death due to microenvironmental cues [6], we choose to update the cellular portion of our model 100 times per cell cycle and refer to this as the cellular automaton timestep, (of duration $\tau/100$) and scale the rates for cell behaviour accordingly (reduced by a factor of 100) so as not to affect the timescale of division. Thereby reducing the oxygen calculations to $2,304$ updates per cellular automaton timestep.

Sensitivity analysis. To assay the model for sensitivity to parameters, we measure the cellularity and cellular-oxygen distributions for the regular vascularity example reported in the centre panel of Figure 2, with $\Theta = 0.0027$ (or a regular spacing of 14 cell diameters). From the parameter set modelled in Figure 2 ($D_c = 0.1$, $r_c = 1$, $K_m = 0.01$and $c_{ap} = 0.1$) we vary each parameter by $\approx$ three orders of magnitude and report the ranges for each mode of cellular oxygen distribution and cellularity in Table S1.

As expected, increasing cellular oxygen consumption (r_c) strongly influences the ability for a given vascular architecture to support cells, with greater consumption correlating with decreased cellularity. The mean oxygen concentration experienced by cells goes down and then up slightly as the number of cells decreases drastically. Variation in the diffusion coefficient, D_c, strongly affects the ability for a domain to support cells, and also the mean and skewness of the resulting oxygen distribution, but affects the standard deviation relatively little. Our choice of threshold for apoptosis, c_{ap}, intuitively has a strong effect on the cellularity, and then inversely on mean cellular oxygen as fewer and fewer cells are competing for the same oxygen. The standard deviation is affected little, and the skewness decreases at first and then increases as the number of cells becomes smaller and smaller.

Spatial statistics. To measure the variation away from homogeneity, we utilise a measure derived from Ripley's K function. To begin, we have

$$\hat{K}(r) = \lambda^{-1} \sum_{i \neq j} \frac{\mathrm{I}(d_{ij} < r)}{n}, \tag{4}$$

where λ is the average density of points in the domain, I is the indicator function which yields

$$\mathrm{I}(d_{ij} < r) = \begin{cases} 1 & \text{if the Euclidian distance between vessels } i \text{ and } j \text{ is less than } r, \\ 0 & \text{otherwise.} \end{cases} \tag{5}$$

Table 1. Sensitivity analysis.

Parameter	Value	Cellularity	Mean cellular-oxygen	Std cellular-oxygen	Skewness cellular-oxygen
D_c					
	0.001	0.0015	0.5198	0.0286	-0.333
	0.01	0.0449	0.4623	0.0916	0.0197
	0.1	0.4336	0.2346	0.1029	1.5947
	0.2	0.8489	0.1931	0.0917	2.2165
r_c					
	0.01	0.9969	0.9796	0.0023	2.2728
	0.1	0.9969	0.7969	0.0227	2.273
	1	0.4336	0.2346	0.1029	1.5947
	10	0.0517	0.3398	0.1296	0.0338
	20	0.0265	0.3716	0.1458	-0.3105
K_m					
	0.001	0.398	0.2705	0.1018	1.4904
	0.01	0.4338	0.2346	0.1029	1.5947
	0.1	0.6862	0.1817	0.0926	2.2194
	0.2	0.9448	0.1702	1.0872	2.494
	0.5	0.9969	0.2816	0.0772	2.3861
c_{ap}					
	0.001	0.8674	0.054	0.0924	2.8366
	0.01	0.6088	0.0849	0.1016	2.3506
	0.1	0.4336	0.2346	0.1029	1.5847
	0.2	0.3495	0.3668	0.0952	1.495
	0.5	0.1883	0.6399	0.0617	1.634

We utilize the variance stabilized version of this measure, $\hat{L}(r)$, which is given by

$$\hat{L}(r) = \frac{\hat{K}(r)}{\pi}^{1/2}, \quad (6)$$

which has an expected value of $\hat{L}(r) = 1$ for homogeneous data. To correct for edge effects, we implement the correction suggested by Ripley [7], which changes the value of the indicator function, for points assayed within r of the edge, to the reciprocal of the proportion of the circle (of radius r) which falls outside the study area.

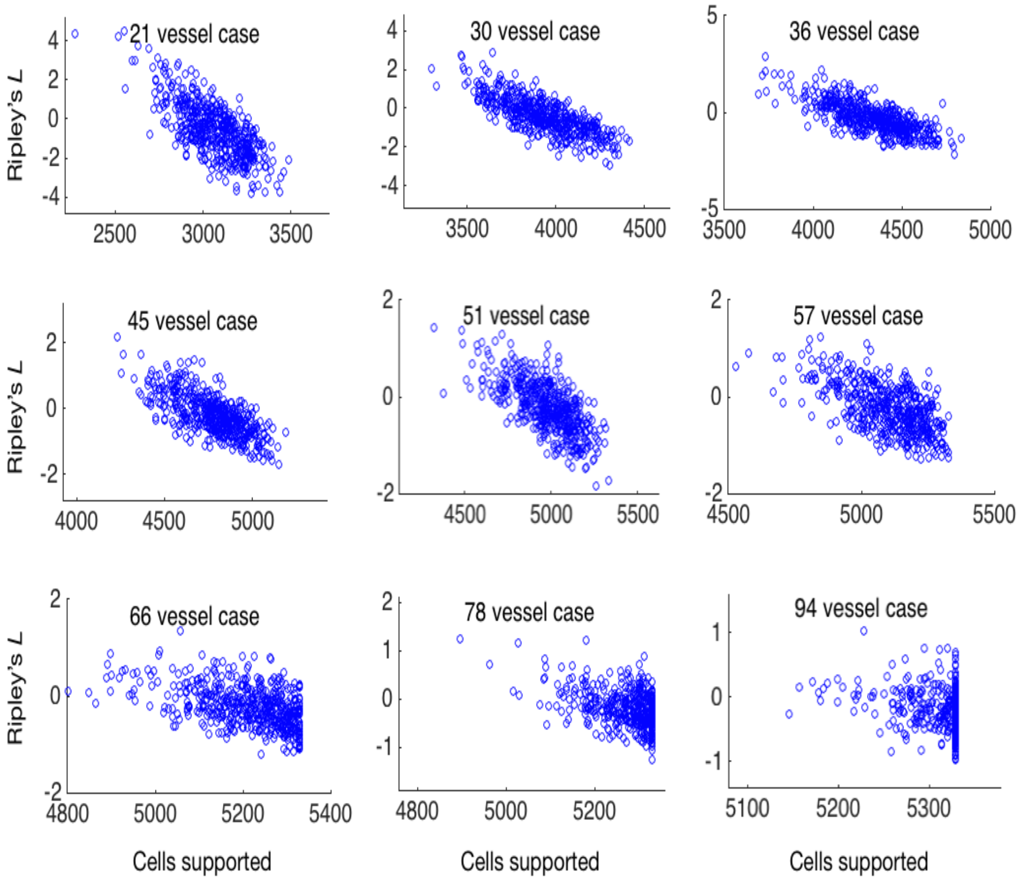

Figure B: Ripley's L function versus carrying capacity. We plot nine scatter plots showing the relationship in each of the 500 simulations represented in Fig. 5 for a given initial vessel density between cell number at equilibrium (x-axis) and Ripley's L (y-axis). We find that there is a significant negative correlation in the low vessel densities which loses predictive capability as the domain becomes entirely filled and all of the data points align at the full carrying capacity.

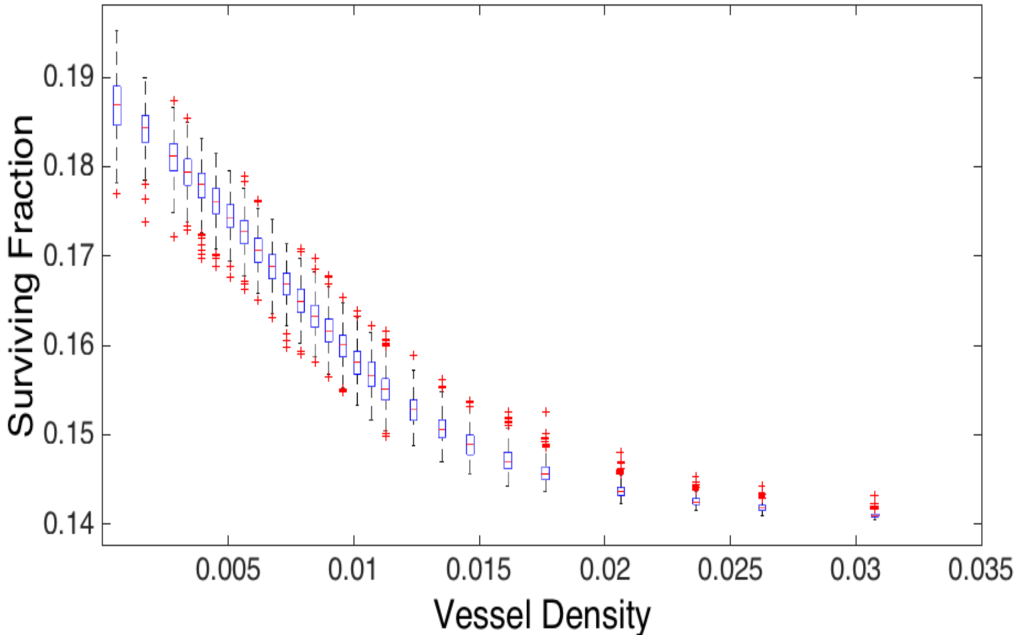

Figure C: Surviving Fraction after 2 Gy as a function of vessel density. We plot the surviving fraction of cells after 2Gy of simulated radiation in each simulation as calculated using, from the main text, equation (5) modified by the OER from equations (6) and (7) versus the number of vessels in each case for each of the 500 simulations with constant vessel number, but random placement, on domain size 73×73 at dynamic equilibrium. The edges of the boxes represent the 25th and 75th percentile, the whiskers extend to the most extreme data points not considered outliers. Outliers are defined as any simulation outside approximately 2.7 standard deviations, and they are plotted as red crosses. We note that the mean is monotonically decreasing, as would be expected from the increasing mean oxygen, but also that this does not capture the dynamic interplay between supported tissue size and vascular architecture, and does not tell us about tumour control probability.

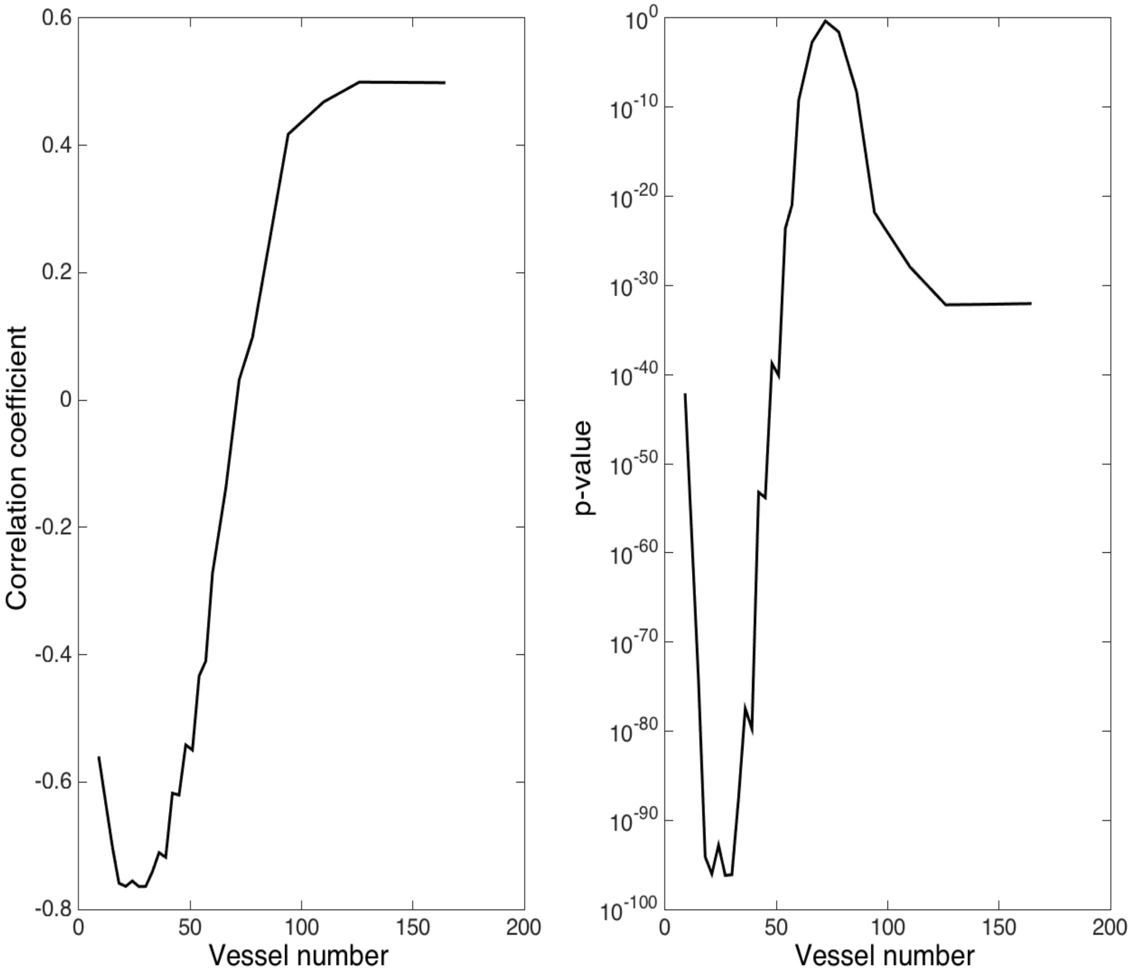

Figure D: Correlation and p-value for Ripley's *L* function vs. surviving cells after radiation. Here we plot the correlation coefficient (Left) vs. vessel density for all families of simulations and the corresponding p-value (Right). We notice that the correlation coefficient changes sign at 70 vessels, and the p-value briefly rises to insignificant (≈ 0.5) at the time of the sign change.

References

1. Carreau A, Hafny-Rahbi BE, Matejuk A, Grillon C, Kieda C. Why is the partial oxygen pressure of human tissues a crucial parameter? Small molecules and hypoxia. J Cell Mol Med. 2011;15(6):1239–1253.
2. Anderson ARA. A hybrid mathematical model of solid tumour invasion: the importance of cell adhesion. Math Med Biol. 2005;22(2):163.
3. Frieboes HB, Edgerton ME, Fruehauf JP, Rose Felicity RAJ, Worrall LK, Gatenby RA, Ferrari M and Cristini, V. Prediction of drug response in breast cancer using integrative experimental/computational modeling. Canc. Res. 2009;69(10):4484–4492.
4. Freyer JP, Tustanoff E, Franko AJ, Sutherland RM. *In situ* oxygen consumption rates of cells in V-79 multicellular spheroids during growth. J Cell Physiol. 1984;118(1):53–61.
5. Mitchell, A.R. and Griffiths, D. F. The finite difference method in partial differential equations. . A Wiley-Interscience Publication, Chichester: Wiley, 1980.
6. Saraste, A. and Pulkki, K. Morphologic and biochemical hallmarks of apoptosis. Cardiovasc. Res. 2000;3(45):528–537.
7. Ripley BD. The second-order analysis of stationary point processes. J Appl Prob. 1976;p. 255–266.
